# Treatment decision for impacted mandibular third molars: Effects of cone-beam computed tomography and level of surgeons’ experience

**DOI:** 10.1371/journal.pone.0314883

**Published:** 2024-12-05

**Authors:** Emine ADALI, Meltem OZDEN YUCE, Gözde IŞIK, Elif ŞENER, Ali MERT

**Affiliations:** 1 Private DCT Clinic- Oral and Dental Health Center, Mugla, Turkey; 2 Department of Oral and Maxillofacial Surgery, School of Dentistry, Ege University, Izmir, Turkey; 3 Department of Oral Radiology, School of Dentistry, Ege University, Izmir, Turkey; 4 Department of Statistics, School of Science, Ege University, Izmir, Turkey; Justus Liebig University Giessen, GERMANY

## Abstract

The aim of this study was to analyse the effect of surgeons’ experience and the benefit of using cone-beam computed tomography (CBCT) images, compared to the use of panoramic radiography (PAN) images, on their decisions with regard to mandibular third molar treatment modality. Panoramic radiographs and CBCTs from a total of 143 patients who had undergone impacted third molar surgery were randomly evaluated for treatment decision by 10 participants with differing clinical experience (5 novices and 5 experienced surgeons). The degree of agreement between the same type of participants was ’Substantial Agreement’ (0.61–0.80) or ’Almost Perfect’ (0.81–1.00). When the treatment modality decisions of the experienced and novice surgeons, using PAN and CBCT images, were compared, a statistically significant difference was found (p<0.01) between the variables. In 50 cases, experienced surgeons decided from CBCT images that total extraction was the best treatment method but opted for coronectomy when presented with PAN images of the same cases. In 164 cases novice surgeons decided on total extraction from CBCT images but chose coronectomy when presented with PAN images of the same cases. The results obtained from this study revealed that the degree of professional experience of the surgeon was a significant factor in determining the treatment decision. Moreover, the treatment choice of experienced surgeons was less affected by the change in imaging technique employed, when compared to novice surgeons.

## Introduction

The surgical removal of impacted third molars is a routine dental operation carried out in oral and maxillofacial surgery practice either for therapeutic or prophylactic purposes [1]. Although it is generally considered a safe procedure, several possible complications may be encountered during the perioperative and postoperative periods. These include the risk of pain, swelling, localised osteitis, post-operative infection, bleeding, jaw fracture, trismus and nerve damage [2]. One serious complication which must be guarded against is inferior alveolar nerve injury (IANI). This complication can cause functional problems and, on some occasions can lead to medico-legal consequences [1, 3]. This may arise due to insufficient diagnosis or due to the use of incorrect surgical technique to remove the mandibular third molar. Therefore, additional radiographic diagnosis is strongly recommended to reduce as far as possible the incidence of complications [2, 4].

In clinical practice, intraoral and panoramic images are considered sufficient to evaluate the relationship between IAN and mandibular third molar apices. This is the standard, recognized diagnostic imaging method for routine cases. Although panoramic radiography (PAN) is the standard tool for preoperative diagnosis, these radiographs provide limited information about the bucco-lingual direction of the tooth roots and the absence of cortical bone in the mandibular canal [5–7]. The development of new imaging technologies such as cone-beam computed tomography (CBCT) has led to great advances in presurgical planning [4, 8, 9]. When compared with panoramic radiographies, CBCT provides better quality images of the teeth and the neighbouring structures by generating a three-dimensional volumetric image in the axial, sagittal and coronal planes with high resolution, no magnification and no superimposition [1, 5]. Also, distortion in CBCT is negligible as it varies between 0.05 and 0.04 mm [2]. Therefore, presurgical CBCT imaging has become an essential routine nowadays.

In this light, the aim of this study was to analyse the effect of surgeons’ experience, and the additional benefit of CBCT images, on their choice of treatment modality decisions made with regard to mandibular third molar. The hypothesis was that the use of preoperative CBCT imaging influences the treatment decisions taken by oral surgeons, when compared to the use of PAN imaging.

## Materials and methods

**Fig 1:**
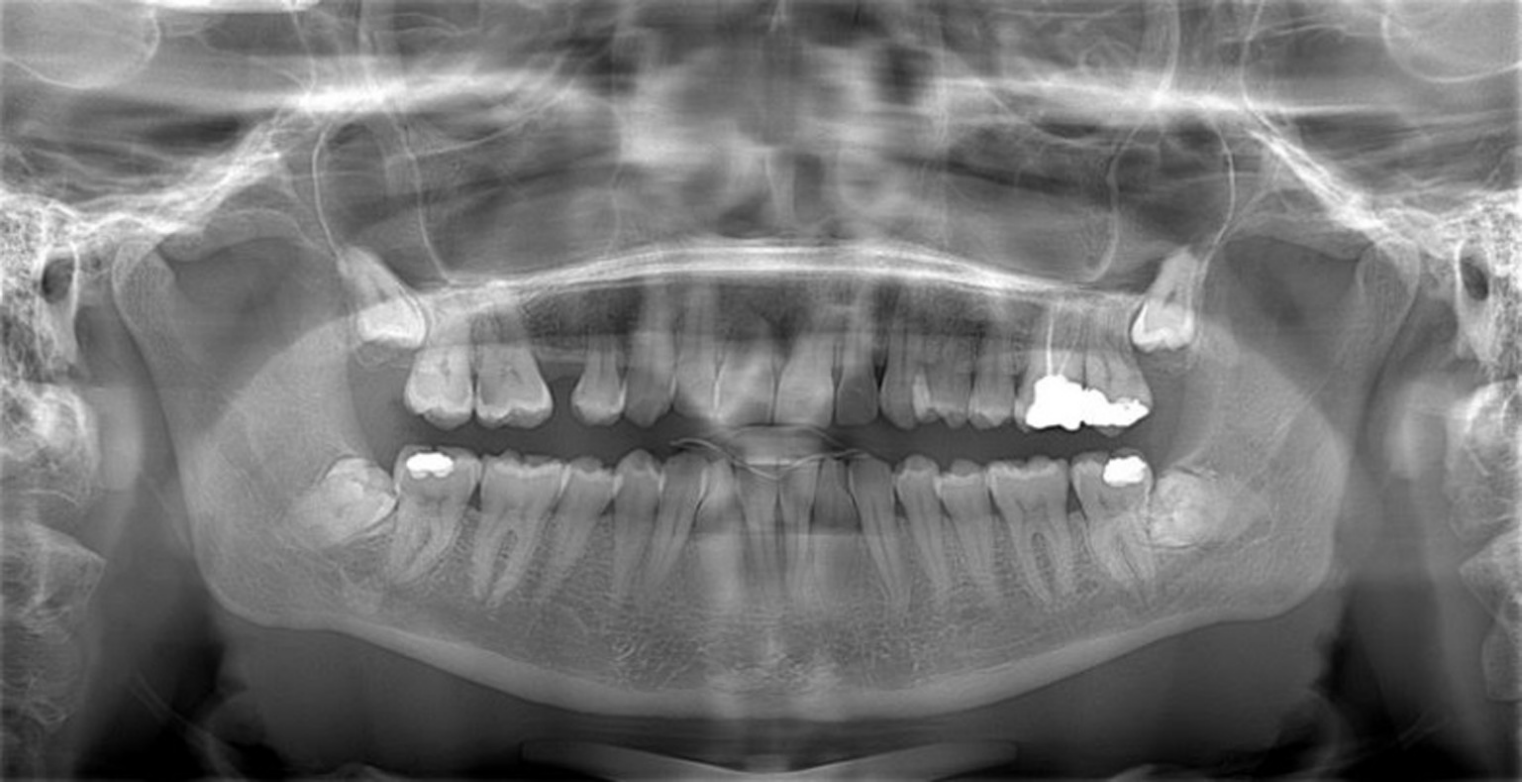
A panoramic image.

**Fig 2:**
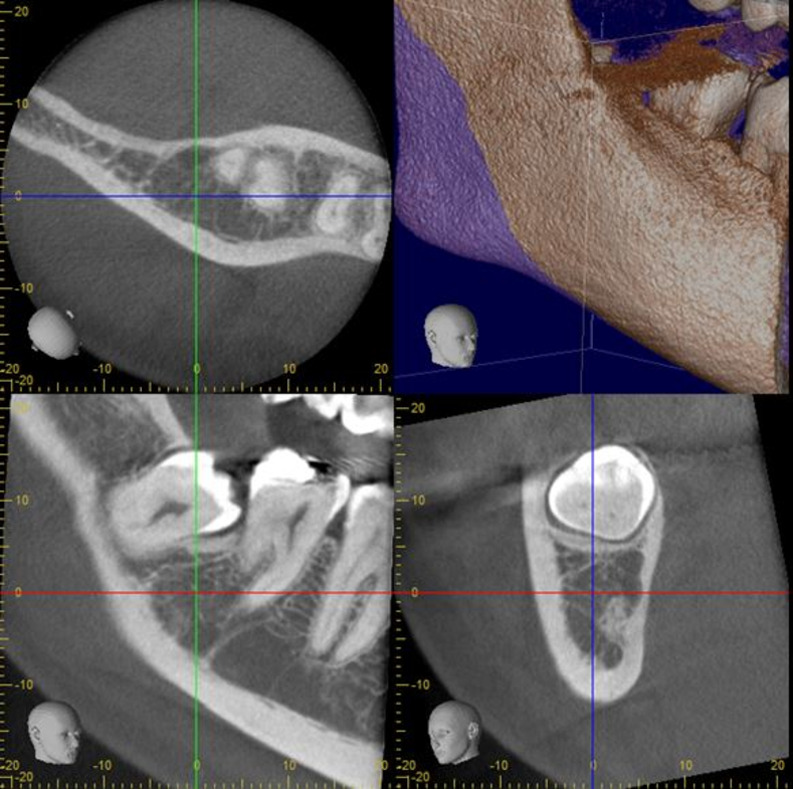
An image of a patient’s CBCT planes.

The study consisted of 150 patients who had undergone impacted third molar surgery and who had received both panoramic radiography and CBCT as part of their regular treatment before the operation. Therefore, both panoramic radiographs and CBCTs were available from the records of each patient. The study was conducted in accordance with the Declaration of Helsinki and the study protocol was approved by the Ethics Committee of the Medical Faculty of the University (20–6.1T/48). The study was started on 23 August 2020 and took two years to collect images and evaluated them by specialists. Images were excluded from the study if the quality was not of sufficiently high standard. Other reasons for exclusion were if the mandibular third molar had incomplete root formation or if there was root displacement due to pathology such as a cyst or tumour. A total of 7 patients were excluded due to the poor quality of panoramic radiography and consequently a total of 143 patients were finally included in the study. A power analysis was conducted using the G*Power program (Statistical Power Analyses for Windows; Dusseldorf, Germany). to determine the required number of experts to evaluate the images in the present study. For this analysis, the inter-rater agreement kappa value from the study by Manor et al. was used [2]. The calculations indicated that, to achieve 95% power in the study, the sample size per group needs to be 4 participants. In the study, 143 panoramic (Fig 1) and 143 CBCT images (Fig 2) were randomly evaluated by two groups of oral surgeons with differing levels of clinical experience (5 novices (0–5 years of seniority) and 5 experienced (more than 5 years of seniority)) from the same faculty. All participants signed consent forms that included details of study. All the images were coded before evaluation and the radiographic images of each patient were evaluated independently and separately by the same surgeon. The participants were asked to decide on their preferred treatment modality for the impacted third molar: whether to perform a coronectomy or a total extraction based on the CBCT and panoramic images. Overall, we collected 1430 treatment decisions from 5 experienced surgeons (715 for PAN images and 715 for CBCT images) and 1430 treatment decisions from 5 novice surgeons (715 for PAN images and 715 for CBCT images).

### Statistical analysis

Statistical analysis was carried out to find out if there was any change to treatment decisions following the CBCT findings and also whether treatment decisions differed between novice and experienced surgeons. Statistical software (SPSS Inc. version 21 IBM, Chicago, IL) was used to analyse the data. The interobserver agreement was calculated as a kappa score. Categorical variables were analyzed using the Chi-square Test. Correlations were determined using Pearson-Spearman tests. Statistical significance level was set at p<0.05.

## Results

In this study, 5 novice surgeons (3 female, 2 male) with a median of 2.1 years seniority and 5 experienced surgeons (2 female, 3 male) with a median of 10,2 years seniority examined 143 PAN and 143 CBCT images. The treatment modality decisions of the novice and experienced surgeons according to the PAN and CBCT images are demonstrated in Table 1. Interobserver agreement between the experienced surgeons, calculated as a weighted kappa score, was 0.89 (range: 0.80–0.97) in relation to treatment decisions using PAN images and 0.93 (range: 0.88–1.00) using CBCT images. Between novice surgeons, inter-observer agreement calculated as a weighted kappa score was 0.85 (range: 0.76–0.96) with regard to treatment decisions using PAN images and 0.74 (range: 0.66–0.85) using CBCT images. Consequently, the degree of agreement between the same type of participant was found to be ’Substantial Agreement’ (0.61–0.80) or ’Almost Perfect’ (0.81–1.00).

**Table 1:** Treatment modality decision of surgeons according to panoramic radiography and CBCT images.

	*Treatment decision according to PAN images*		*Total*	*Treatment decision according to CBCT images*		*Total*
***Surgeons***	Coronectomy	Total Extraction		Coronectomy	Total extraction	
Experienced	91 (%13)	624 (%87)	715	43 (%6)	672 (%94)	715
Novice	268 (%37)	447 (%63)	715	127 (%18)	588 (%82)	715

When the treatment modality decisions of the experienced surgeons from the PAN and CBCT images were compared using the Chi-square test, a statistically significant difference was found (p<0.01). In 50 cases, experienced surgeons decided on total extraction when presented with the CBCT images but opted for coronectomy from the PAN images. However, there were only 2 cases where they decided on coronectomy from the CBCT images but chose total extraction after viewing the PAN images. Moreover, a significant difference was found between experienced and novice surgeons in determining treatment modality from the PAN images (p<0.01). Novice surgeons decided on coronectomy from PAN images in 179 cases which the experienced surgeons decided required total extraction (Table 2). When the treatment decisions of the novice surgeons from the PAN and CBCT images were compared using the chi-square test, a statistically significant difference was found (p<0.01). Novice surgeons decided on total extraction from the CBCT images in 164 cases which they determined from the PAN images required coronectomy. Also, there was a statistically significant difference between experienced and novice surgeons in determining the treatment method with regard to the CBCT images (p<0.01). Novice surgeons decided on coronectomy in 86 cases which experienced surgeons determined from the CBCT images required total tooth extraction (Table 3).

**Table 2:** Comparison of surgeons’ treatment decisions based on imaging method.

		*Experienced surgeons’ treatment decision according to CBCT images*			*Novice surgeons’ treatment decision according to PAN images*		
		Coronectomy	Total Extraction	Total	Coronectomy	Total extraction	Total
***Experienced surgeons’ treatment decision according to PAN images***	Coronectomy	41	50	91	89	2	91 (%13)
	Total extraction	2	622	624	179	445	624 (%87)
Total		43 (%6)	672 (%94)	715	268 (%37)	447 (%63)	715
*Chi-Square Test*		*P<0*.*01/ significant difference between groups*					

**Table 3:** Comparison of surgeons’ treatment decisions based on imaging method.

		*Experienced surgeons’ treatment decision according to CBCT images*			*Novice surgeons’ treatment decision according to PAN images*		
		Coronectomy	Total Extraction	Total	Coronectomy	Total extraction	Total
***Novice surgeons’ treatment decision according to CBCT images***	Coronectomy	41	86	127	104	23	127 (%18)
	Total extraction	2	586	588	164	424	588 (%82)
Total		43 (%6)	672 (%94)	715	268 (%37)	447 (%63)	715
*Chi-Square Test*		*P<0*.*01/ significant difference between groups*					

The correlation observed between the treatment modality decisions of experienced surgeons based on CBCT and PAN images was moderate, r = 0.63 (p<0.05). This demonstrates that the treatment choice of experienced surgeons is affected by the change in imaging technique employed less than novice surgeons. Also, there was found to be a moderate correlation (0.51) between the novice and experienced surgeons’ treatment choices based on the CBCT images (p<0.05) (Table 4).

**Table 4:** Correlations coefficient according to Pearson-Spearman tests.

	*Experienced surgeons’ treatment decision according to PAN images*	*Novice surgeons’ treatment decision according to PAN images*	*Experienced surgeons’ treatment decision according to CBCT images*
***Novice surgeons’ treatment decision according to PAN images***	0,48		
***Experienced surgeons’ treatment decision according to CBCT images***	0,63	0,33	
***Novice surgeons’ treatment decision according to CBCT images***	0,50	0,43	0,51
*Pearson-Spearman Test*	p<0.05/ significant difference between groups		

## Discussion

The results of this study showed that the degree of professional experience of the surgeon was a significant factor in determining the treatment decision. Although, experienced surgeons were less affected than the novices by the change of imaging method, the use of preoperative CBCT imaging was found to affect the treatment decisions of experienced and novice surgeons, when compared to the use of PAN. Therefore, our hypothesis was accepted.

The management of third molars is the most widely researched subject in oral and maxillofacial surgery, as with many surgical procedures andit can give rise to a variety of complications [4]. IANI is one of these complications which can significantly affect a patient’s quality of life. This neurological complication may develop as a result of insufficient preoperative radiological diagnosis and/or deficient technique in performing the surgery [1]. In addition, the extent of the surgeon’s experience, and the effect of traumatic tissue manipulation with excessive and uncontrolled force, can be counted as etiologic factors associated with damage to IAN [1, 10–12]. Sisk et al. [13] reported that the age and experience of the surgeons were significant factors with regard to such complications as alveolar osteitis and nerve dysesthesia. In their study, the incidence of IANI was found to be nine times greater in cases treated by less-experienced surgeons [13]. Similarly, according to Jerjes et al., [10] patients treated by trainees have a higher prevalence of nerve dysesthesia [10]. Moreover, the sensory disturbance in the IAN after third molar surgery can be eliminated in high-risk cases if operations are planned carefully [14]. According to the literature, if the relationship between the roots of an impacted third molar and the IAN is not clear on the PAN, then additional 3D radiography is critical for accurate preoperative assessment. This assists the surgeon in determining the risk of IANI before surgery, and the surgeon can then modify the surgical technique used [2, 15].

CBCT findings seem to offer more advantages, especially to novice surgeons, by giving them the confidence to opt for total extraction in most of the cases. Coronectomy has become popular in high-risk cases where direct contact between the mandibular third molar and the mandibular canal is observed by radiographic examination. With this technique, the crown is sectioned and the roots that are closer to the IAN are left in situ. If there is a high risk of IANI, it is generally recommended that, coronectomy should be the treatment choice to minimize the risk of damage to the IAN [16]. Mukherjee et al. [17] reported that coronectomy is an effective procedure in controlling IAN damage in radiographically-evaluated high-risk cases and they found a very low incidence of complications. Broken fragments of vital teeth are generally thought to heal without complications [17, 18]. Because of this low complication rate and being regarded as a safer treatment modality, coronectomy has generally recognized as the preferred method for high-risk cases. In our study, when the treatment decisions of novice surgeons using PAN and CBCT images were compared, a statistically significant difference was found. If there was direct contact between the tooth and the mandibular canal, novice surgeons preferred coronectomy to protect the IAN, and they decided total extraction was required in 164 cases from the CBCT images but opted for coronectomy for the same cases from the PAN images.

Camargo et al. [4] assessed the treatment modalities of the surgeons according to their experience. They reported that experienced surgeons tended to manage mandibular third molars based on PAN images only, and they chose coronectomy as a treatment choice twice as frequently in the case of high-risk patients [4]. In their study, Manor et al. [2] reported that the use of CBCT prior to mandibular third molar extraction has little effect on the treatment decision taken by the experienced surgeon [2]. This report agrees with Matzen et al. [19] who reported that CBCT did not change treatment decisions according to PAN in 88% of patients, only in 12% of the cases did the treatment decision change after CBCT [19]. Our study results confirmed these findings. Compared to the novice surgeons, the experienced surgeons decided on total extraction only in 50 cases but opted for coronectomy for the same cases from the PAN images.

The limitation of the present study is that the number of researchers included is not large enough to be clinically significant. Therefore, further research is recommended to evaluate more fully the effect of surgeons’ experience on treatment decisions with regard to impacted mandibular third molars.

## Conclusion

In conclusion, the results obtained from this study revealed that the degree of professional experience of the surgeon was a significant factor in determining the mandibular third molar treatment modality. Also, experienced surgeons were less affected than the novices by the change of imaging method. Surgeons feel more confident when CBCT images are available since they feel better able to make accurate treatment decisions. Therefore, further studies with large sample sizes will be required to better understand the effect of surgeons’ experience and the benefit of using CBCT images on treatment decisions.

## Supporting information

S1 File Relevant data. (XLSX)
